# Supplementary material for: Quality Indicators Compliance and Survival Outcomes in Breast Cancer according to Age in a Certified Center
Source: Cancers (Basel). 2023 Feb 24;15(5):1446. doi: 10.3390/cancers15051446 (PMC10000816; doi:10.3390/cancers15051446)
Supplement: Supplementary file 1 [file cancers-15-01446-s001.zip › Supplementary Fig.S2.pdf]

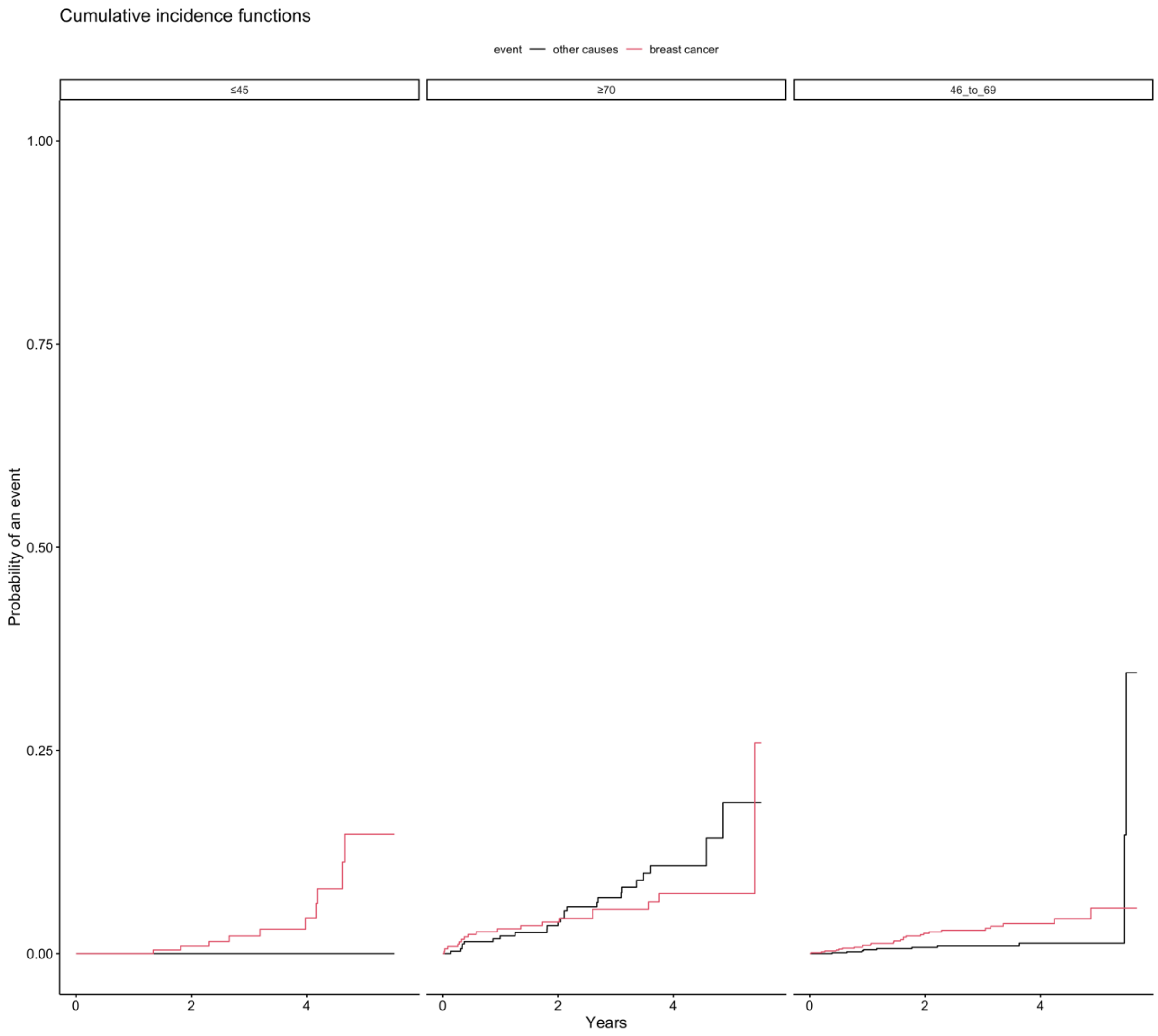

Supplementary Figure S2. Competitive risk curves. Death from all causes ($p < 0.001$), Death from breast cancer ($p < 0.089$).
