# Supplementary material for: Quality Indicators Compliance and Survival Outcomes in Breast Cancer according to Age in a Certified Center
Source: Cancers (Basel). 2023 Feb 24;15(5):1446. doi: 10.3390/cancers15051446 (PMC10000816; doi:10.3390/cancers15051446)
Supplement: Supplementary file 1 [file cancers-15-01446-s001.zip › Supplementary Table S1.pdf]

Supplementary Table S1. Patient's prognosis stratified by age group.

Characteristic	N	≤45y, N = 310[1]	46-69y, N = 926[1]	≥70y, N = 344[1]	p-value[2]
Local recurrence (yes)	1,580	2 (0.6%)	15 (1.6%)	5 (1.5%)	0.5
Regional recurrence (yes)	1,580	2 (0.6%)	4 (0.4%)	0 (0%)	0.4
Distant metastases (yes)	1,580	16 (5.2%)	51 (5.5%)	16 (4.7%)	0.8
Site of distant metastases	83				0.3
Brain		3 (19%)	5 (9.8%)	1 (6.2%)	
Liver		4 (25%)	5 (9.8%)	1 (6.2%)	
Other lymphnodes		0 (0%)	4 (7.8%)	0 (0%)	
Multiple sites		3 (19%)	11 (22%)	3 (19%)	
Bone		4 (25%)	20 (39%)	7 (44%)	
Lung		0 (0%)	5 (9.8%)	1 (6.2%)	
Other		2 (12%)	1 (2.0%)	3 (19%)	
Patient status	1,580				<0.001
Died		10 (3.2%)	36 (3.9%)	42 (12%)	
Alive with progression of disease		10 (3.2%)	34 (3.7%)	11 (3.2%)	
Alive with no evidence of disease		290 (94%)	856 (92%)	291 (85%)	
Cause of death	88				<0.001
Breast cancer		10 (100%)	26 (72%)	18 (43%)	
Other causes		0 (0%)	10 (28%)	24 (57%)	

[1]n (%)

[2]Fisher's Exact Test for Count Data with simulated p-value (based on 2000 replicates); Pearson's Chi-squared test
