# Supplementary material for: Quality Indicators Compliance and Survival Outcomes in Breast Cancer according to Age in a Certified Center
Source: Cancers (Basel). 2023 Feb 24;15(5):1446. doi: 10.3390/cancers15051446 (PMC10000816; doi:10.3390/cancers15051446)
Supplement: Supplementary file 1 [file cancers-15-01446-s001.zip › Supplementary Table S2.pdf]

	All causes (crude)					Breast cancer (crude)				
Characteristic	N	Event N	HR[1]	95% CI[1]	p-value	N	Event N	HR[1]	95% CI[1]	p-value
Age group	1,580	88				1,580	54			
46-69y			—	—				—	—	
≤45y			0.83	0.41, 1.68	0.6			1.16	0.56, 2.42	0.7
≥70y			3.41	2.18, 5.32	<0.001			2.02	1.11, 3.69	0.022
Tumor size	1,551	83	1.03	1.02, 1.03	<0.001	1,551	51	1.03	1.02, 1.04	<0.001
Chemotherapy (yes)	1,415	83	0.28	0.14, 0.55	<0.001	1,415	54	0.34	0.15, 0.75	0.007
Radiotherapy (yes)	1,580	88	0.27	0.18, 0.42	<0.001	1,580	54	0.26	0.15, 0.45	<0.001
Surgery (yes)	1,580	88	0.07	0.05, 0.12	<0.001	1,580	54	0.05	0.03, 0.09	<0.001
Axillary status	1,415	83				1,415	54			
Negative			—	—				—	—	
Positive			4.13	2.68, 6.37	<0.001			8.69	4.95, 15.3	<0.001
Modified Bloom-Richardson Grade	1,415	83				1,415	54			
G1			—	—				—	—	
G2			1.66	0.87, 3.17	0.13			2.35	0.87, 6.39	0.093
G3			2.23	1.19, 4.19	0.013			4.76	1.85, 12.2	0.001

[1]HR = Hazard Ratio, CI = Confidence Interval

Table S2: Cox proportional hazards progression model.
