# Supplementary material for: Quality Indicators Compliance and Survival Outcomes in Breast Cancer according to Age in a Certified Center
Source: Cancers (Basel). 2023 Feb 24;15(5):1446. doi: 10.3390/cancers15051446 (PMC10000816; doi:10.3390/cancers15051446)
Supplement: Supplementary file 1 [file cancers-15-01446-s001.zip › Supplementary Table S3.pdf]

Supplementary Table S3- Adjusted Standardized Residuals Pearson's Chi-squared

**Characteristic**	**≤45y**	**46-69y**	**≥70y**
Sex			
Woman	0.9	-1.5	0.9
Man	-0.9	1.5	-0.9
BMI (group)			
Not evaluated	**-7.1**	-1.7	**8.8**
< 18.5 - 24.9	**9.7**	-2.6	**-6.2**
25.0 - 29.9	0.1	3.3	**-4.0**
≥ 30.0	-1.7	2.1	-0.9
Referral from screening programme			
1	**-9.0**	**13.9**	**-7.8**
2	**9.0**	**-13.9**	**7.8**
Clinical examination/Suspicious of malignancy			
No	**-4.7**	**6.6**	-3.3
Yes	**4.7**	**-6.6**	3.3
Side location of the lesion			
Left	-0.6	1.2	-0.9
Right	0.6	-1.2	0.9
Disease extent by imaging or clinical examination			
Localized	**-4.1**	0.8	3.0
Multicentric/Multifocal	**4.1**	-0.8	-3.0
MRI			
No	**-12.5**	0.7	**11.2**
Yes	**12.5**	-0.7	**-11.2**
Axillary staging (incl. only invasive tumours)			

**Characteristic**	**≤45y**	**46-69y**	**≥70y**
Negative	**-3.6**	2.8	0.2
Positive	**3.6**	-2.8	-0.2
TNM stage			
0	-1.9	2.6	-1.3
I	**-3.1**	**3.7**	-1.5
II	**4.2**	**-4.7**	1.6
III	**3.0**	**-3.6**	1.4
IV	-2.4	1.4	0.6
Invasive histological type at biopsy (incl. invasive and microinvasive tumours)			
Ductal NST	1.6	0.5	-2.1
Lobular	-1.2	0.3	0.8
Other	-1.0	-0.9	2.0
Final pathology			
In situ	-1.9	2.6	-1.3
Invasive (incl. microinvasive)	-3.6	-0.9	**4.6**
Invasive at biopsy only with pathological complete response	**7.5**	-1.6	**-5.3**
Grade (incl. only invasive tumours)			
I	**-4.4**	1.6	2.3
II	-2.6	0.0	2.5
III	**6.5**	-1.4	**-4.6**
Presence or absence of lymphovascular invasion in operated invasive tumors			
Not evaluated	**-3.8**	**-5.2**	**9.6**
No	-2.7	**5.0**	-3.3
Yes	**5.5**	-2.1	-2.6
Oestrogen receptor status (incl. only invasive tumours)			

Characteristic	≤45y	46-69y	≥70y
Not performed	2.0	-1.2	-0.5
Negative	2.3	-0.9	-1.1
Positive	-2.4	1.0	1.1
Progesterone receptor status (incl. only invasive tumours)			
Not performed	-1.6	0.2	1.4
Negative	0.0	0.6	-0.7
Positive	0.3	-0.6	0.5
Her2 overexpression (incl. only invasive tumours) (with immunohistochemistry)			
Not Performed	-2.2	**-5.0**	**8.0**
Negative	-2.1	1.3	0.6
Positive	2.9	0.2	-3.0
Proliferation activity (Ki67) in grade 2 invasive tumours			
Not performed	-1.7	-3.2	**5.1**
<5%	-0.4	1.7	-1.5
5-30%	0.9	2.9	**-4.1**
>30%	2.5	-0.4	-1.7
Molecular (incl. only invasive tumours)			
LUMA	-2.7	1.7	0.7
LUMB	2.1	0.0	-2.0
HER2 MISSING	-2.2	**-5.0**	**8.0**
HER2	1.5	0.1	-1.5
TN	1.6	-0.7	-0.7
BRCA1 + BRCA2			
No genetic assessment	**-18.6**	**7.1**	**9.5**
Negative	**17.9**	**-6.8**	**-9.1**

Characteristic	≤45y	46-69y	≥70y
Positive	**3.9**	-1.5	-2.0
First treatment			
Surgery	**-4.1**	**7.0**	**-4.4**
Neoadjuvant chemotherapy	**9.2**	-1.0	**-7.7**
Primary hormonotherapy	**-5.5**	**-9.0**	**16.1**
Palliative treatment/Surveillance/Patient refusal	-1.6	-1.8	**3.7**
Surgery			
Breast conservative surgery	**-4.0**	**6.2**	-3.5
Mastectomy	**6.3**	**-3.7**	-1.7
No surgery	**-4.3**	**-5.5**	**10.6**
Endocrine therapy			
No	0.6	-0.1	-0.5
Yes	-0.6	0.1	0.5
Chemotherapy			
No	-2.3	**-3.8**	**6.8**
Yes	2.3	**3.8**	**-6.8**
Radiotherapy			
No	-0.4	**-7.0**	**8.7**
Yes	0.4	**7.0**	**-8.7**

Values in bold are those driving the significance reported in Table 1.

To ensure a conservative significance assessment, we accounted for all multiple comparisons in the table. To accomplish this, we used the Bonferroni correction to adjust the alpha significance level from 0.05 to a corrected p-value of 0.00012, which corresponds to a critical value of N (0,1) = 3.7. Consequently, any values greater than 3.7 in absolute terms are considered significantly different from what we would expect under the null hypothesis.
