# Supplementary figures and images for: Quality Indicators Compliance and Survival Outcomes in Breast Cancer according to Age in a Certified Center
Source: Cancers (Basel). 2023 Feb 24;15(5):1446. doi: 10.3390/cancers15051446 (PMC10000816; doi:10.3390/cancers15051446)

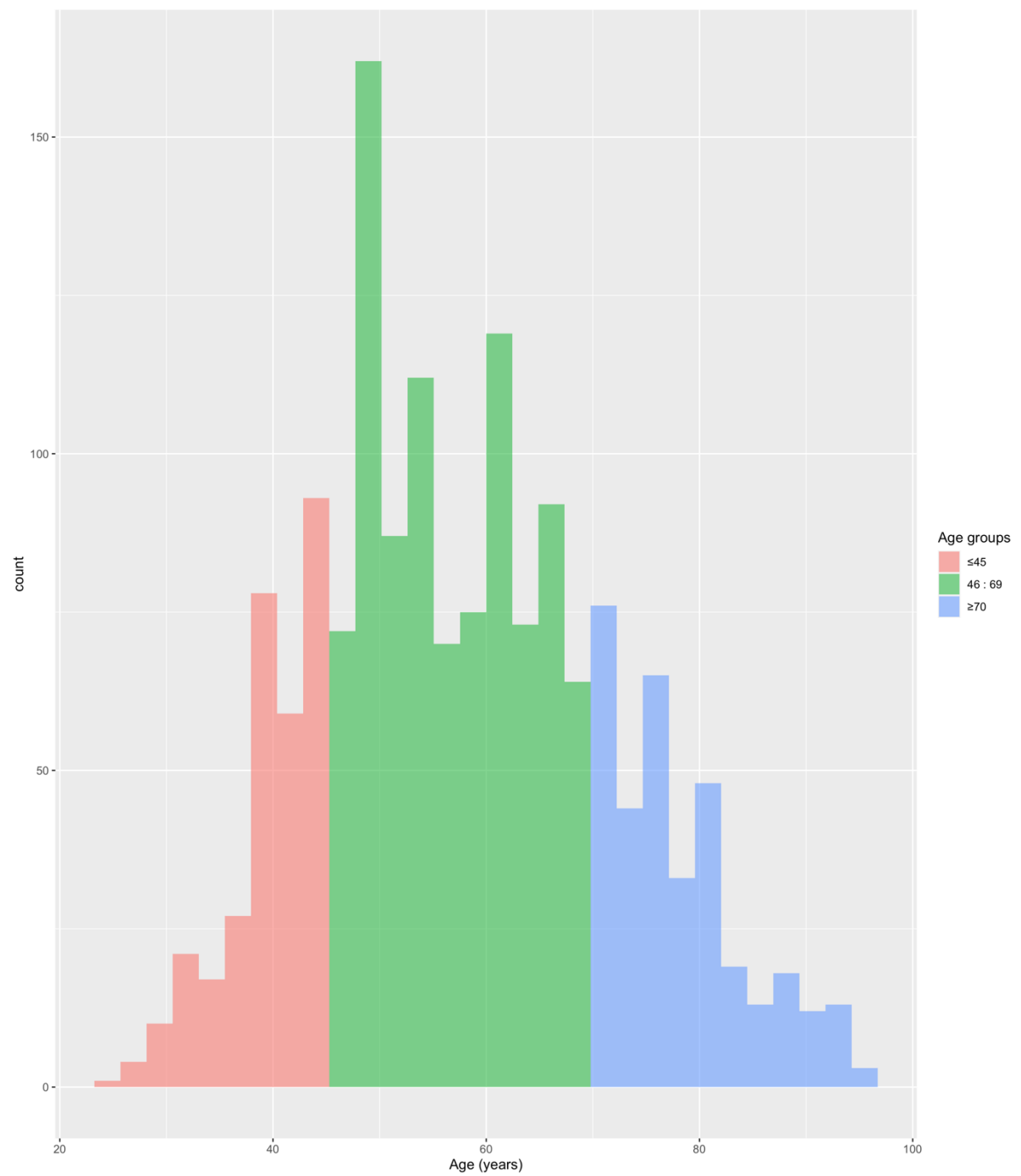

Supplementary Figure S1. Histogram and descriptive statistics of age groups.

Supplement: Supplementary file 1 [file cancers-15-01446-s001.zip › Supplementary Fig.S1.pdf]

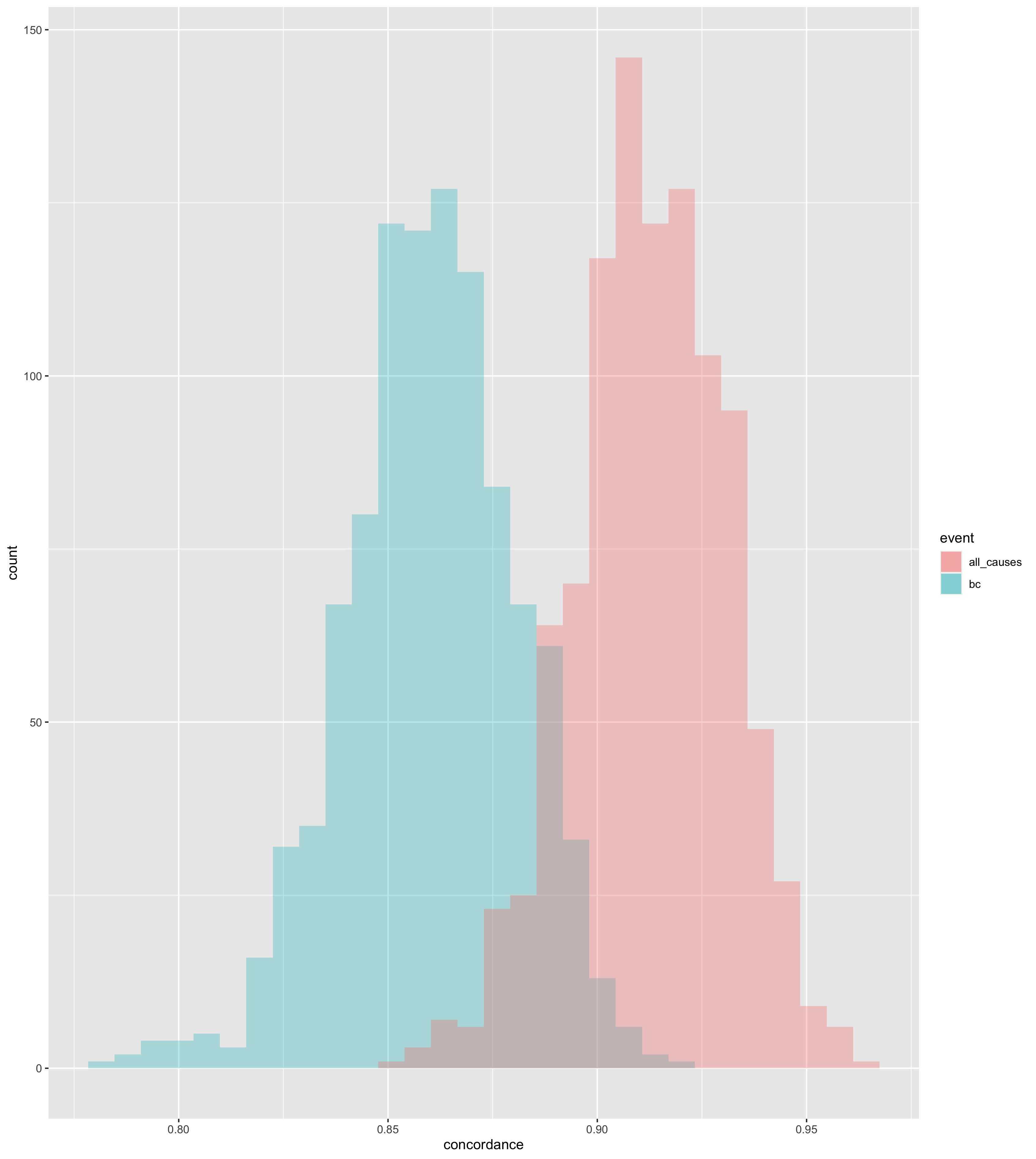

Supplement: Supplementary file 1 [file cancers-15-01446-s001.zip › Supplementary Fig.S3.png]
